# Clinical features of COVID-19 in Italian outpatient children and adolescents during Parental, Delta, and Omicron waves: a prospective, observational, cohort study

**DOI:** 10.3389/fped.2023.1193857

**Published:** 2023-08-10

**Authors:** Costanza Di Chiara, Riccardo Boracchini, Giulia Sturniolo, Alessia Barbieri, Paola Costenaro, Sandra Cozzani, Marica De Pieri, Cecilia Liberati, Annachiara Zin, Andrea Padoan, Francesco Bonfante, Fatima Kakkar, Anna Cantarutti, Daniele Donà, Carlo Giaquinto

**Affiliations:** ^1^ Division of Pediatric Infectious Diseases, Department for Women’s and Children’s Health, University of Padua, Padua, Italy; ^2^ Penta – Child Health Research, Padua, Italy; ^3^ Division of Biostatistics, Epidemiology and Public Health, Laboratory of Healthcare Research and Pharmacoepidemiology, Department of Statistics and Quantitative Methods, University of Milano-Bicocca, Milan, Italy; ^4^ Department of Medicine-DIMED, University of Padua, Padua, Italy; ^5^ Division of Comparative Biomedical Sciences, Istituto Zooprofilattico Sperimentale delle Venezie, Padua, Italy; ^6^ Division of Infectious Diseases, Department of Pediatrics, CHU Sainte-Justine, Montréal, QC, Canada

**Keywords:** COVID-19, SARS-CoV-2, clinical manifestations, symptoms, pediatric population, children, variants of concern

## Abstract

**Introduction:**

COVID-19 features changed with the Omicron variant of SARS-CoV-2 in adults. This study aims to describe COVID-19 symptoms in children and adolescents during the Parental, Delta, and Omicron eras

**Methods:**

A single-centre, prospective observational study was conducted on individuals aged 0–20 years attending the University Hospital of Padua (Italy) from April 2020 to December 2022. COVID-19 cases were defined by positive SARS-CoV-2 molecular detection and/or serology; patient/family symptoms and virological positivity were considered to determine the infection onset. Variables were summarized and compared using appropriate tests of descriptive statistics

**Results:**

A total of 509 cases [46% female, median age eight years (IQR: 4–12)] were studied. Three-hundred-eighty-seven (76%), 52 (10%), and 70 (14%) subjects experienced COVID-19 during the Parental, Delta, and Omicron waves, respectively. All subjects developed an asymptomatic/mild COVID-19. Overall, the most frequent symptoms were fever (47%) and rhinitis (21%), which showed a significant increasing incidence from the Parental to Omicron waves (*p* < 0.001). Conversely, diarrhea was most common during the pre-Omicron eras (*p* = 0.03). Stratifying symptoms according to the age group, fever, rhinitis, and skin rashes were observed more frequently among infants/toddlers; conversely, fatigue was more common in children older than five years. The duration of symptoms was similar across different SARS-CoV-2 variants of concern (VOCs); conversely, the number of symptoms varied according to the age group (*p* < 0.0001)

**Discussion:**

This study showed differences in COVID-19 clinical presentation among infants, children, and adolescents and confirmed Omicron infection is more likely to be associated with upper respiratory symptoms. However, further population-based studies are needed to support these findings. In addition, active surveillance will play a crucial role in assessing the disease severity of future VOCs.

## 1. Introduction

SARS-CoV-2, like other RNA viruses, is inclined to evolve with the development of mutations over time, resulting in the emergence of multiple variants that may have different genetic features compared to its ancestral strains, reflecting different virulence and clinical manifestations (1). Since January 2022, the Omicron (B.1.1.529) variant of concern (VOC) has replaced other variants in Italy, accounting for more than 95% of the total cases in the Veneto Region (2). This new variant and its sublineages demonstrated higher transmissibility, with a significant increase in the number of pediatric SARS-CoV-2 infections compared to the previous waves, leading to an increased incidence of hospitalization in the pediatric population (3, 4). However, several studies have documented lower severity and mortality rates following the Omicron infection than earlier variants in both adults and children, as well as a decreased incidence of Multisystem Inflammatory Syndrome in Children (MIS-C) (5–7).

The emergence of new SARS-CoV-2 variants and the questionable differences in COVID-19 clinical manifestations among VOCs in children remains poorly understood. Moreover, since late 2021, other seasonal respiratory viruses have reappeared, and the co-circulation of SARS-CoV-2 with other viruses that cause flu-like syndromes has led to an increasing clinical diagnostic challenge due to the similarity of acute symptoms (8, 9). To date, most of the studies describing the clinical impact of SARS-CoV-2 VOCs have been conducted in adults, and those including children are limited to the early months of the Omicron wave (3, 10–12).

We are currently facing the seventh pandemic wave, and children represent more than 18% of all cases (13). Moreover, SARS-CoV-2 started to spread simultaneously with other seasonal respiratory viruses that can lead to flu-like syndromes in Italy (14). Hence, a greater knowledge of the clinical presentation spectrum of COVID-19 in children is crucial to help pediatricians in their clinical practice at the community level, providing them with a tool to recognize SARS-CoV-2 early from other respiratory infections.

Finally, while some of these (influenza and rotavirus) are vaccine preventable, little is known about vaccination uptake during these different pandemic waves, and how, if at all, vaccination may mitigate the symptoms of SARS-CoV-2 infection in children.

This study aims to describe the epidemiological and clinical features of SARS-CoV-2 infection due to the Parental, Delta, and Omicron variants in a prospective outpatient cohort of COVID-19 family clusters affected mainly by the asymptomatic or mild disease recruited at the Department of Women's and Children's Health of Padua University Hospital.

## 2. Materials and methods

### 2.1. Setting and data collection

We conducted a single-centre, prospective cohort study on children and adolescents who attended the COVID-19 Family Cluster Follow-up Clinic (CovFC) between April 2020 and December 2022. The CovFC is an outpatient multidisciplinary pediatric clinic that has been established in March 2020 at the Department of Women's and Children's Health, University Hospital of Padua (Veneto region, Italy), with the aim of evaluating and following families from the Veneto region after a household outbreak of SARS-CoV-2. Families were referred to the CovFC by their family pediatricians (FPs) 8–12 weeks after the end of isolation if meeting the following criteria: (a) having at least a child in the pediatric age (0≤15 years old); (b) having one or more family member/s with a history of COVID-19 virologically-confirmed by a positive nasal-pharyngeal swab (NPS).

At enrolment, a pediatrician collected data on demographic parameters, past medical history (15, 16), and routine and COVID-19 immunization status from all family members, and performed a clinical evaluation of the child/children. Moreover, a blood sample was collected from all family members for the detection of anti-receptor binding domain (RBD) antibodies against SARS-CoV-2 spike protein [MAGLUMI™2000 Plus, Snibe Diagnostics, Snibe Diagnostics, New Industries Biomedical Engineering Co., Ltd (Snibe), Shenzhen, China], and/or the quantification of SARS-CoV-2 neutralizing antibodies with a high throughput method for Plaque Reduction Neutralization Test (PRNT) (15, 16).

For each confirmed COVID-19 case, the severity of the disease was clinically scored as mild, moderate, severe, critical, or MIS-C following the World Health Organization (WHO) classification (17). Subsequently, COVID-19 confirmed cases underwent a 6-monthly clinical and serological follow-up for at least one year after the infection. All data collected at enrollment and during each follow-up visit were anonymized and entered into a web-based database using the REDCap® platform (Vanderbilt University, Tennessee). As of March 2023, the CovFC cohort included approximately 480 families, for a total of 1,500 pediatric and adult outpatients. The study protocol was approved by the local Ethics Committee (Prot. N° 0070714 of 24th November 2020; last amendment Prot. N° 0024018 del 5/4/2022). Parents or legally authorized representatives were informed of the research proposal and provided written consent to use the routine patient-based data for research purposes.

### 2.2. Study design, case identification, and exposure definition

Among the patients enrolled in the CovFC, all children and older siblings of 0–≤20 years old were considered COVID-19 confirmed cases and were included in the analysis if they met the following criteria: (1) had a record of virological positivity for SARS-CoV-2 by RT-PCR and/or (2) had a positive serological test performed before receiving COVID-19 vaccination.

For each individual enrolled in the study, a baseline date of infection was defined by two blinded pediatricians with expertise in the field of pediatric infectious diseases as follows: (1) for symptomatic cases: the date of symptoms onset or the date of first positive antigenic or molecular SARS-CoV-2 NPS; (2) for asymptomatic cases: the date of the first positive NPS or, in those with only serologically confirmed COVID-19 and with negative/undetermined NPS, by the family outbreak, coinciding with the date of infection onset in the family cluster. Where discordant classification occurred, records were verified by a third blinded pediatrician. Subjects who were asymptomatic and had no analytical evidence of SARS-CoV-2 infection were considered non-COVID-19 cases and were excluded from the analysis.

COVID-19 cases were classified according to the predominant circulating SARS-CoV-2 VOC in the Veneto Region at the time of children's infection onset (baseline date) using the CovSPECTRUM platform, which is based on surveillance data (2). The following main VOCs were considered for the analysis: Parental, Delta, and Omicron (including B.1.1.529, BA.2, BA.4, and BA.5 sublineages). Any SARS-CoV-2 infection that occurred in the Veneto region from February 2020 to 14 June 2021, had a probability greater than 96% to be caused by Parental VOC; any SARS-CoV-2 infection that occurred in the Veneto region from 14 July 2021, to 11 December 2021, had a probability greater than 96% to be caused by Delta VOC; any SARS-CoV-2 infection that occurred in the Veneto region from 7 January 2022, to 8 December 2022, had a probability greater than 96% to be caused by the Omicron VOC. COVID-19 cases exposed to SARS-CoV-2 infection out of these three predefined pandemic waves were excluded from the analysis.

### 2.3. Statistical analysis

Clinical and sociodemographic characteristics were summarized according to the number, percentage, median and interquartile range (IQR), as appropriate, stratified according to the VOCs. To assess the different clinical manifestations of the SARS-CoV-2 infection in children over the pandemic waves, the *χ*^2^ or Fisher exact test and Wilcoxon's test were used for categorical and continuous variables, respectively. The Cochran–Armitage test for trend was used to capture changes in symptoms moving from the Parental to Omicron waves. In addition, to assess the impact of age (stratified to 0–2, 3–4, 5–11, 12–20 years), underlying disease (i.e., the presence of at least one comorbidity), routine childhood vaccinations, and influenza and rotavirus vaccinations, on the clinical manifestations of COVID-19 in children, we applied a sub-stratification for each variable of interest to the main one (i.e., by VOC). Duration of symptoms was obtained as the difference between the end of the last symptom and the onset of the first one. All the statistical analyses were performed using the Statistical Analysis System Software (version 9.4; SAS Institute, Cary, NC, USA).

## 3. Results

### 3.1. Demographic features of the study population

From 1 April 2020, to 8 December 2022, 438 family clusters of COVID-19 were evaluated at the CovFC resulting to a total of 757 adult parents, and 717 children and adolescents. Among children and adolescents, one hundred and twenty-five individuals (17.4%) were classified as non-COVID-19 cases and were excluded from the study. Among the 592 pediatric COVID-19 cases, 83 were excluded as they had a baseline date out of the predefined periods, in addition to 9 who were aged more than twenty years (Supplementary Figure S1). Four-hundred and thirty-six (85.7%) subjects tested positive for SARS-CoV-2 by antigen or molecular NPS, and 73 (14.3%) individuals, antigen negative, but having either one of the two serological tests used in this investigation, were included in the study as COVID-19 cases (Supplementary Figure S1). All 73 COVID-19 cases diagnosed through the presence of anti-S-RBD and/or neutralizing antibodies were in individuals who experienced SARS-CoV-2 infection during the Parental wave when COVID-19 vaccines were not yet accessible.

As a result, 509 pediatric COVID-19 cases were studied [234 (46%) females, overall median age of 8 years (IQR: 4.38–11.53)], including 387 (76%), 52 (10.2%), and 70 (13.8%) individuals who experienced infection during the Parental, Delta, and Omicron VOC waves, respectively. Overall, 139 children (27%) had an asymptomatic infection and 370 children (73%) had a mild COVID-19. No cases were classified as moderate/severe or critical. MIS-C complicated eleven (2%) cases; of which, 9 (81.8%), 1 (9.1%), and 1 (9.1%) were diagnosed during the Parental, Delta, and Omicron VOCs waves, respectively. SARS-CoV-2 infection was more frequent in subjects older than five, representing 71% (*N* = 360) of the entire study population. One hundred and nineteen individuals (23%) had at least one previous underlying disease (Table 1); asthma was the most common. Finally, only 30 (5.9%) subjects received at least one dose of mRNA COVID-19 vaccine before infection or during follow-up (Table 1).

**Table 1:** Sociodemographic and clinical characteristics of the study population (*N *= 509), overall and according to the SARS-CoV-2 variant of concern (VOC).

	Parental (*N* = 387)	Delta (*N* = 52)	Omicron (*N* = 70)	Total (*N* = 509)
Age in years, median (P25–P75)	8.3 (4.37–11.87)	7.18 (4.84–10.11)	7.32 (4.09–9.8)	8.08 (4.38–11.53)
0–2 years	67 (17.31)	8 (15.38)	10 (14.29)	85 (16.70)
3–4 years	47 (12.14)	7 (13.46)	10 (14.29)	64 (12.57)
5–11 years	165 (42.64)	29 (55.77)	37 (52.86)	231 (45.38)
12–20 years	108 (27.91)	8 (15.38)	13 (18.57)	129 (25.34)
Gender, *N* (%)				
Female	178 (45.99)	23 (44.23)	33 (47.14)	234 (45.97)
Male	209 (54.01)	29 (55.77)	37 (52.86)	275 (54.03)
Number of symptoms, *N* (%)				
0	124 (32.04)	13 (25)	2 (2.86)	139 (27.31)
1	110 (28.42)	10 (19.23)	14 (20)	134 (26.33)
2	69 (17.83)	14 (26.92)	21 (30)	104 (20.43)
≥3	84 (21.71)	15 (28.85)	33 (47.14)	132 (25.93)
Vaccinations status^b^, *N* (%)				
Without primary vaccinations	54 (14.36)	6 (11.76)	17 (24.64)	77 (15.52)
Primary vaccinations	322 (85.64)	45 (88.24)	52 (75.36)	419 (84.48)
Rotavirus vaccines^a^, *N* (%)				
No	119 (31.32)	10 (19.61)	19 (27.14)	148 (29.54)
Yes	261 (68.68)	41 (80.39)	51 (72.86)	353 (70.46)
Flu vaccines 19–21^c^, *N* (%)				
No	286 (85.12)	22 (88)	19 (76)	327 (84.72)
Yes	50 (14.88)	3 (12)	6 (24)	59 (15.28)
COVID vaccination^d^, *N* (%)				
First Dose	4 (1.78)	2 (5.26)	24 (48)	30 (9.58)
Second Dose	0 (0)	0 (0)	1 (100)	1 (100)
Comorbidities^e^, *N* (%)				
0	303 (78.29)	41 (78.85)	46 (65.71)	390 (76.62)
At least 1	84 (21.71)	11 (21.15)	24 (34.29)	119 (23.38)

^a^ Patients aged at least 61 days.

^b^ Patients aged at least 3 months, 13 missing values are handled in the analysis.

^c^ Patients aged at least 6 months, 94 missing values are handled in the analysis.

^d^ Patients aged at least 5 years, 50 & 362 missing values are handled in the analysis respectively for first and second dose.

^e^ Among 509 individuals, prematurity (*N *= 8), obesity (*N *= 2), diabetes mellitus (*N *= 2), asthma (*N *= 11), congenital heart disease (*N *= 4), rheumatic conditions (*N *= 3), nephropathy (*N *= 3), neurologic disorders (*N *= 4), chronic hepatitis (*N *= 3), other comorbidities (*N *= 92). “Other comorbidities” included conditions that were not classified as risk factors for severe COVID-19 (15, 16), such as respiratory and food allergies, atopic dermatitis, recurrent urinary tract infections, hearing loss, irritable bowel syndrome and inflammatory bowel diseases, celiac disease, dermatological diseases, febrile seizure, behavior alterations, and periodic fever.

### 3.2. Clinical features of COVID-19 overall and according to the SARS-CoV-2 VOC

We described the clinical features of SARS-CoV-2 infection in the entire study population (*N* = 509). Three-hundred and seventy (73%) individuals developed a symptomatic infection, of which 134 (26%), 104 (20%), and 132 (26%) had 1, 2, and ≥3 symptoms, respectively. Overall, fever (*N* = 238, 46.8%), rhinitis (*N* = 105, 20.6%), headache (*N* = 80, 15.7%), fatigue (*N* = 79, 15.5%), and cough (*N* = 75, 14.7%) were the most frequent symptoms, followed by hyposmia and/or ageusia (*N* = 46, 9%) and diarrhea (*N* = 39, 7.7%) (Table 2). To better evaluate the role of each VOC in the clinical manifestation of SARS-CoV-2 infection, COVID-19 cases were stratified according to the Parental, Delta, and Omicron VOCs. An increasing incidence of fever (*p* < 0.0001) and upper respiratory tract symptoms, such as rhinitis (*p* < 0.0001), cough (*p* < 0.0001), and sore throat (*p* = 0.0045), were observed from the Parental to Omicron VOC waves. Moreover, systemic symptoms, including myalgia (*p* = 0.027) and poor feeding (*p* = 0.047), and gastrointestinal symptoms, including nausea and vomiting (*p* = 0.033), were more frequently caused by the Omicron VOC. On the other hand, “other symptoms”, including elevation of body temperature between 37 and 37.4°C, tachycardia, chest pain, restlessness, vertigo, photophobia, and chilblains, were less common during the Delta VOC wave (*p* = 0.034).

**Table 2:** Frequency of COVID-19 clinical manifestations, trend of symptoms’ changes from the Parental to the Omicron waves, and symptoms duration in the study population (*N *= 509), overall and according to the different VOC.

	Overall (*N* = 509)	Parental (*N* = 387)	Delta (*N* = 52)	Omicron (*N* = 70)	*p*-value^a^	*p*-Trend^a^
Symptoms, *N* (%)						
Rhinitis	105 (20.63)	62 (16.02)	17 (32.69)	26 (37.14)	<0.0001	< 0.0001
Cough	75 (14.73)	37 (9.56)	14 (26.92)	24 (34.29)	<0.0001	< 0.0001
Dyspnea	7 (1.38)	5 (1.29)	0 (0)	2 (2.86)	0.1010	0.4687
Fever	238 (46.76)	158 (40.83)	27 (51.92)	53 (75.71)	<0.0001	< 0.0001
Otalgia	2 (0.39)	1 (0.26)	1 (1.92)	0 (0)	0.1557	0.8075
Myalgia	21 (4.13)	14 (3.62)	2 (3.85)	5 (7.14)	0.0275	0.2031
Arthralgia	15 (2.95)	13 (3.36)	0 (0)	2 (2.86)	0.0558	0.5428
Sore throat	28 (5.50)	17 (4.39)	1 (1.92)	10 (14.29)	0.0004	0.0045
Hyposmia and/or ageusia	46 (9.04)	38 (9.82)	5 (9.62)	3 (4.29)	0.3277	0.1691
Conjunctivitis	12 (2.36)	10 (2.58)	1 (1.92)	1 (1.43)	0.1213	0.5323
Fatigue	79 (15.52)	55 (14.21)	9 (17.31)	15 (21.43)	0.2871	0.1148
Headache	80 (15.72)	56 (14.47)	8 (15.38)	16 (22.86)	0.2068	0.0939
Alteration of consciousness	0 (0)	0 (0)	0 (0)	0 (0)	–	–
Abdominal pain	15 (2.95)	13 (3.36)	1 (1.92)	1 (1.43)	0.0841	0.3293
Nausea/vomiting	23 (4.52)	16 (4.13)	2 (3.85)	5 (7.14)	0.0338	0.3206
Diarrhea	39 (7.66)	31 (8.01)	4 (7.69)	4 (5.71)	0.8018	0.5270
Poor feeding	20 (3.93)	15 (3.88)	1 (1.92)	4 (5.71)	0.0472	0.6419
Lymphadenopathy	1 (0.20)	1 (0.26)	0 (0)	0 (0)	0.7603	0.5970
Skin rash	12 (2.36)	11 (2.84)	1 (1.92)	0 (0)	0.0594	0.1491
Pneumonia	3 (0.59)	2 (0.52)	0 (0)	1 (1.43)	0.2393	0.4814
Other symptoms^b^	35 (6.88)	28 (7.24)	2 (3.85)	5 (7.14)	0.0348	0.7681
Symptoms duration, days	3 (1–6)	3 (1–6)	2.5 (2–7)	2.5 (1–4)	0.2499	

^a^ –*χ*^2^ or Fisher as appropriated.

^b^ “Other symptoms” included elevation of body temperature between 37 and 37.4°C, tachycardia, chest pain, restlessness, vertigo, photophobia, and chilblains.

Overall, symptoms lasted for a median of 3 days (IQR: 1–6), without differences according to the different VOCs [3 (IQR: 1–6), 2.5 (IQR: 2–7), and 2.5 (IQR: 1–4) days during the Parental, Delta, and Omicron VOC waves, respectively (*p* = 0.25)].

Among 30 subjects who were immunized against SARS-CoV-2, 23 (77%) received COVID-19 vaccination before getting infected by the Omicron VOC. Focusing on those individuals who got SARS-CoV-2 during the Omicron wave (*N* = 70), fever was more common in unvaccinated individuals (*N* = 47) compared to previously immunized children (*N* = 23) (*p* = 0.042). Moreover, unvaccinated children recorded more symptoms compared to immunized subjects (*p* = 0.017) (Supplementary Table S1).

To better assess the impact of age on the clinical features of COVID-19, we compared symptoms stratifying subjects across four age groups (0–2, 3–4, 5–11, and 12–20 years of age) (Table 3). We observed that some symptoms differ among age groups. Rhinitis (*p* = 0.042), fever (*p* = 0.002), and diarrhea (*p* = 0.092) were more frequent in individuals younger than two years of age. In addition, poor feeding (*p* = 0.0002) and abdominal pain (*p* = 0.0009) were more common in children aged 0–2 and 3–4 years, respectively. On the other hand, nausea/vomiting was more frequently reported by adolescents aged 12–20 years (*p* = 0.0045). Furthermore, myalgia (*p* = 0.0002), arthralgia (*p* = 0.0009), sore throat (*p* < 0.0001), hyposmia and/or ageusia (*p* < 0.0001), fatigue (*p* = 0.027), and headache (*p* < 0.0001) were observed more frequently among individuals aged 5–11 and 12–20 years (Table 3). To better explore the clinical symptoms of COVID-19 in infants and young children, we stratified patients into five different age groups (0–2 months, 3–11 months, 1–4 years, 5–11 years, and 12–20 years) (Supplementary Table S2). Among infants aged 1–2 months, the most common symptoms observed were rhinitis, fever, and poor feeding. However, in older infants aged 3–11 months, diarrhea was more prevalent. Abdominal pain was not identified in subjects under 1 year of age, while nausea and vomiting were found to be more common in subjects older than 5 years. A similar pattern of symptoms was observed stratifying COVID-19 cases according to the Parental, Delta, and Omicron VOC (Supplementary Table S3). Conversely, no significant differences were observed in the number and duration of symptoms according to the age group (Table 3).

**Table 3:** Clinical features of COVID-19 in the study population (*N *= 509) among different age groups (0–2, 3–4, 5–11, and 12–20 years).

	0–2 years	3–4 years	5–11 years	12–20 years	*p*-value
Symptoms, *N* (%)					
Rhinitis	25 (29.41)	15 (23.44)	36 (15.58)	29 (22.48)	0.0426
Fever	54 (63.53)	31 (48.44)	91 (39.39)	62 (48.06)	0.002
Myalgia	0 (0)	1 (1.56)	10 (4.33)	10 (7.75)	0.0002
Arthralgia	0 (0)	3 (4.69)	5 (2.16)	7 (5.43)	0.0009
Sore throat	1 (1.18)	1 (1.56)	13 (5.63)	13 (10.08)	<0.0001
Hyposmia and/or ageusia	0 (0)	1 (1.56)	19 (8.23)	26 (20.16)	<0.0001
Fatigue	5 (5.88)	8 (12.5)	40 (17.32)	26 (20.16)	0.0274
Headache	0 (0)	3 (4.69)	48 (20.78)	29 (22.48)	<0.0001
Abdominal pain	0 (0)	4 (6.25)	9 (3.9)	2 (1.55)	0.0009
Nausea/vomiting	3 (3.53)	1 (1.56)	11 (4.76)	8 (6.2)	0.0045
Diarrhea	12 (14.12)	5 (7.81)	13 (5.63)	9 (6.98)	0.0918
Poor feeding	7 (8.24)	5 (7.81)	6 (2.6)	2 (1.55)	0.0002
Number of symptoms					
0	15 (17.65)	20 (31.25)	76 (32.9)	28 (21.71)	0.1426
1	30 (35.29)	13 (20.31)	58 (25.11)	33 (25.58)	
2	19 (22.35)	14 (21.88)	42 (18.18)	29 (22.48)	
≥3	21 (24.71)	17 (26.56)	55 (23.81)	39 (30.23)	
Duration of symptoms					
*Days*	3 (1–6)	3 (1–7)	2.5 (1–5)	3 (1–7)	0.4714

To assess the impact of underlying disease on the clinical manifestations of COVID-19 in the pediatric population, we evaluated symptoms in previously healthy children compared to those with at least one comorbidity. Among 509 patients, 119 (23%) had at least one comorbidity (Table 1). Overall, we observed that upper and lower respiratory symptoms, including rhinitis (*p* = 0.053), cough (*p* = 0.005), dyspnea (*p* = 0.046), and pneumonia (*p* = 0.012), were more frequent in children with comorbidities compared to healthy individuals (Supplementary Table S4). A similar pattern was observed stratifying patients according to the VOC. Overall, the two groups noticed no differences in the number and duration of symptoms.

### 3.3. Non-SARS-CoV-2 immunization status and COVID-19 clinical manifestation

We investigated the impact of routine childhood vaccinations on COVID-19 clinical features in 496 individuals between 6 months and 20 years of age. Among 509 subjects, 496 (97%) had ≥3 months of life and were included in this analysis. Of these, 419 (85%) were up-to-date with the childhood vaccination; conversely, 77 individuals (16%) were completely unvaccinated (Table 1). Overall, no significant differences in clinical manifestations were observed between vaccinated and unvaccinated individuals, except for diarrhea and “other symptoms” that were more frequent in unvaccinated children (Table 4). There were no differences in the number and duration of symptoms (Table 4).

**Table 4:** Clinical features of COVID-19 between children who were up-to-date vaccinated and those who were not, overall, and stratified according to the VOC.

*Vaccinations*	Overall (*N* = 496)		*p*-value	Parental (*N* = 387)		Delta (*N* = 52)		Omicron (*N* = 70)	
	Non primary	Primary		Non primary	Primary	Non primary	Primary	Non primary	Primary
Symptoms, *N* (%)									
Rhinitis	18 (23.38)	82 (19.57)	0.4442	10 (18.52)	48 (14.91)	3 (50)	14 (31.11)	5 (29.41)	20 (38.46)
Cough	14 (18.18)	59 (14.08)	0.3506	7 (12.96)	29 (9.01)	3 (50)	11 (24.44)	4 (23.53)	19 (36.54)
Dyspnea	1 (1.3)	5 (1.19)	0.4033	1 (1.85)	3 (0.93)	0 (0)	0 (0)	0 (0)	2 (3.85)
Fever	41 (53.25)	187 (44.63)	0.1632	26 (48.15)	123 (38.2)	2 (7.41)	25 (55.56)	13 (76.47)	39 (75)
Otalgia	0 (0)	2 (0.48)	0.7134	0 (0)	1 (0.31)	0 (0)	1 (2.22)	0 (0)	0 (0)
Myalgia	1 (1.3)	20 (4.77)	0.1082	0 (0)	14 (4.35)	0 (0)	2 (4.44)	1 (5.88)	4 (7.69)
Arthralgia	3 (3.9)	12 (2.86)	0.2283	3 (5.56)	10 (3.11)	0 (0)	0 (0)	0 (0)	2 (3.85)
Sore throat	4 (5.19)	24 (5.73)	0.2129	2 (3.7)	15 (4.66)	0 (0)	1 (2.22)	2 (11.76)	8 (15.38)
Hyposmia and/or ageusia	8 (10.39)	38 (9.07)	0.1517	6 (11.11)	32 (9.94)	1 (16.67)	4 (8.89)	1 (5.88)	2 (3.85)
Conjunctivitis	2 (2.6)	9 (2.15)	0.2939	2 (3.7)	7 (2.17)	0 (0)	1 (2.22)	0 (0)	1 (1.92)
Fatigue	7 (9.09)	71 (16.95)	0.0819	6 (11.11)	49 (15.22)	0 (0)	9 (20)	1 (5.88)	13 (25)
Headache	14 (18.18)	66 (15.75)	0.5941	8 (14.81)	48 (14.91)	3 (50)	5 (11.11)	3 (17.65)	13 (25)
Alteration of consciousness	0 (0)	0 (0)	–	0 (0)	0 (0)	0 (0)	0 (0)	0 (0)	0 (0)
Abdominal pain	1 (1.3)	14 (3.34)	0.2182	1 (1.85)	12 (3.73)	0 (0)	1 (2.22)	0 (0)	1 (1.92)
Nausea/vomiting	3 (3.9)	20 (4.77)	0.2310	3 (5.56)	13 (4.04)	0 (0)	2 (4.44)	0 (0)	5 (9.62)
Diarrhea	11 (14.29)	28 (6.68)	0.0227	7 (12.96)	24 (7.45)	1 (16.67)	3 (6.67)	3 (17.65)	1 (1.92)
Poor feeding	3 (3.9)	15 (3.58)	0.2478	3 (5.56)	10 (3.11)	0 (0)	1 (2.22)	0 (0)	4 (7.69)
Lymphadenopathy	0 (0)	1 (0.24)	0.8448	0 (0)	1 (0.31)	0 (0)	0 (0)	0 (0)	0 (0)
Skin rash	1 (1.3)	11 (2.63)	0.2917	1 (1.85)	10 (3.11)	0 (0)	1 (2.22)	0 (0)	0 (0)
Pneumonia	0 (0)	3 (0.72)	0.6022	0 (0)	2 (0.62)	0 (0)	0 (0)	0 (0)	1 (1.92)
Other symptoms	10 (12.99)	24 (5.73)	0.0205	8 (14.81)	19 (5.9)	0 (0)	2 (4.44)	2 (11.76)	3 (5.77)
Number of symptoms, *N* (%)									
0	14 (18.18)	124 (29.59)	0.0550	12 (22.22)	112 (34.78)	2 (3.33)	10 (22.22)	0 (0)	2 (3.85)
1	22 (28.57)	106 (25.3)		17 (31.48)	87 (27.02)	0 (0)	10 (22.22)	5 (29.41)	9 (17.31)
2	23 (29.87)	78 (18.62)		14 (25.93)	52 (16.15)	2 (33.33)	12 (26.67)	7 (41.18)	14 (26.92)
≥3	18 (23.38)	111 (26.49)		11 (20.37)	71 (22.05)	2 (33.33)	13 (28.89)	5 (29.41)	27 (51.92)
Duration of symptoms									
Days	3 (1–7)	3 (1–6)	0.4323	3 (2–7)	3 (1–6)	8 (2–14)	2.5 (2–7)	1 (0–3)	3 (1–4)

A similar analysis was conducted to evaluate the impact of influenza and rotavirus vaccinations on the clinical features of COVID-19 in children. Among 509 subjects, only 59 (15%) were up-to-date with the seasonal flu vaccination. Moreover, only 353 (70%) individuals were fully vaccinated against rotavirus before SARS-CoV-2 infection (Table 1). Stratifying patients according to the influenza and rotavirus vaccinations, no differences in COVID-19 manifestations, number, and duration of symptoms were detected between the two groups, except for rhinitis that was more frequent in children who were immunized for rotavirus compared to those who did not receive rotavirus vaccination (Supplementary Table S5).

## 4. Discussion

In this study, we described the changing clinical features of SARS-CoV-2 infection according to the Parental, Delta, and Omicron VOC in a prospective cohort of Italian infants, children, and adolescents affected by mainly asymptomatic or mild COVID-19. We demonstrated that clinical manifestation of COVID-19 among children and adolescents varies according to the SARS-CoV-2 variant in all age groups, showing an increasing incidence of fever (40.8% vs. 75.7%, *p* < 0.0001) and upper respiratory tract symptoms [including cough (9.6% vs. 34.3%, *p* < 0.0001), rhinitis (16% vs. 37.1%, *p* < 0.0001), and sore throat (4,4% vs. 14.3%, *p* = 0.0045)] from the Parental to Omicron wave.

The uniqueness of this cohort of families allowed for a real-life evaluation of the clinical features of SARS-CoV-2 infection in the pediatric population, exploring differences related to age groups. Moreover, studying family clusters enabled the inclusion of children with a serologically confirmed COVID-19 who experienced an asymptomatic infection. Additionally, being composed of more than 95% of mild COVID-19 cases, this cohort globally reflects the epidemiological situation of SARS-CoV-2 infection (13).

In this study, we confirmed that fever, respiratory symptoms, diarrhea, headache, fatigue, and alteration of smell and taste are the most frequent symptoms of SARS-CoV-2 infection in the pediatric age. Stratifying patients according to the infecting VOC, we found that fever and upper respiratory symptoms, including rhinitis, cough, and sore throat, showed an increasing incidence from the Parental to Omicron waves, confirming that Omicron B.1.1.529 and its sublineages have a particular predilection for the upper respiratory tract also in the pediatric population. Furthermore, we observed that nausea, vomiting, poor feeding, and myalgia were more common during the Omicron wave compared to the Parental and Delta ones. In addition, “other symptoms”, including body temperature between 37 and 37.4°C, tachycardia, chest pain, vertigo, and chilblains, were more represented in children who experienced infection during the Parental and Omicron waves compared to those who got infected by the Delta VOC.

These results align with previous studies documenting a higher incidence of upper respiratory tract symptoms in children and adolescents who experienced SARS-CoV-2 infection during the Omicron era compared to the previous pandemic waves (10, 11, 18, 19). In particular, Tagarro et al. (11), in a cohort study including 109 pediatric outpatients infected in the Omicron era, observed a higher number of fever and upper respiratory tract symptoms and lower cases of pneumonia compared to 546 outpatients during the pre-Omicron waves. In line with these findings, emerging pediatric data describes a significantly higher proportion of croup cases during the Omicron wave than the Delta one in young children (10, 20, 21). The predilection of the Omicron variant for the upper respiratory tract had already been largely documented in adult patients, unlike previous VOCs, which often affected the lower respiratory tract (22, 23).

In addition, in line with our findings, Taytard et al. (12) and Iijima et al. (10) showed a higher frequency of poor feeding and nausea/vomiting during the Omicron compared to the Delta wave.

Conversely, despite previous studies, diarrhea was more frequently recorded during the Omicron era (10–12), our results showed no differences in intestinal manifestations among the three viral variants waves. Similarly, headaches did not appear to vary among different VOCs, contrary to other previous studies (11).

Our results showed that COVID-19 symptoms might vary according to age in the pediatric population. While fever and upper respiratory tract symptoms were more common in infants and young children, on the other hand, myalgia, fatigue, sore throat, headache, and hyposmia/ageusia resulted more frequently in children older than five years of age and adolescents. Similarly, previous authors have found that younger children were more likely to have fever at presentation than older children (24). In addition, previous studies documented that systemic symptoms, such as headache, hyposmia/ageusia, and sore throat were more common in children aged 5–11 years and adolescents (24–26). However, symptoms such as hyposmia/ageusia and headache can be less represented among children younger than 5 years due to the lack of communication of the symptom.

Comparing the clinical features of COVID-19 between vaccinated and unvaccinated subjects, we found a higher frequency of fever among unvaccinated; in addition, unvaccinated children seemed to have more concomitant symptoms compared to vaccinated individuals. Similarly, previous authors showed that vaccinated adults had fewer systemic symptoms, like fever, compared to unvaccinated subjects (27).

The strength of our study is to provide a wide description of the clinical features of COVID-19 according to the VOC in a cohort of children of different ages, affected mainly by an asymptomatic and mild disease that did not require hospitalization. Our findings provide a broad landscape of the dynamic changes in the clinical features of COVID-19 in the general pediatric population during the pandemic. However, our study has several limitations. Firstly, the single-centre type of this study led to the small number of cases, limiting the analysis in the different age groups because of the relatively small sample size; however, to date, a limited number of studies described the clinical features of COVID-19 in children including the first year of the Omicron wave. Moreover, although our data did not show any differences in COVID-19 clinical manifestations between subjects who were up-to-date with routine vaccinations and children who were not, since previous studies documented a cross-reactive T-cell immune response elicited by some vaccines, such as measles-mumps-rubella (MMR) or tetanus-diphtheria-pertussis (Tdap), that mitigate COVID-19 (28, 29). Therefore, our findings should be confirmed by larger cohorts. In addition, the long-term clinical impact of infection due to different variants was not assessed. In the same pediatric cohort, we have previously observed that 157 children and adolescents who experienced an asymptomatic or mild COVID-19 during the Parental and Delta waves showed a significant increase in left ventricular myocardial deformation abnormalities compared to 107 age and body surface area-comparable healthy controls −20.5% ± 2.9% vs. −21.8% ± 1.7%; *p* < 0.001) (30, 31). Moreover, we observed that the incidence of left ventricular myocardial deformation abnormalities persisted higher in COVID-19 cases compared to healthy controls up to 148 days after infection (30, 31). Conversely, in a sub-cohort of individuals with no effect, who experienced infection in the pre-Omicron era, we found no effect on respiratory symptoms and lung function up to 10 months after infection (32). Furthermore, the effects of the SARS-CoV-2 vaccination were not studied, although the relatively small number of subjects of 12–20 years of age presenting with COVID-19 during the Omicron wave might have been imputable to the increased immunization coverage of this age group.

In conclusion, this study showed that there are differences in COVID-19 clinical presentation among infants, children, and adolescents, while the Omicron infection is more likely to be associated with upper respiratory and gastrointestinal symptoms compared to the previous variants. Further population-based studies are needed to support these findings. In addition, active surveillance will play a crucial role in assessing the disease severity of future VOCs, assessing their long-term clinical impact, confirming the effectiveness of vaccines, and evaluating the clinical impact of other respiratory viruses on SARS-CoV-2 co-infection severity and outcomes.

## Data Availability

The raw data supporting the conclusions of this article will be made available by the authors, without undue reservation.
