# Supplementary material for: Clinical features of COVID-19 in Italian outpatient children and adolescents during Parental, Delta, and Omicron waves: a prospective, observational, cohort study
Source: Front Pediatr. 2023 Aug 10;11:1193857. doi: 10.3389/fped.2023.1193857 (PMC10450148; doi:10.3389/fped.2023.1193857)
Supplement: Supplementary file 1 [file Datasheet1.pdf]

Supplementary Material

Clinical Features of COVID-19 in Italian Outpatient Children and Adolescents During Parental, Delta, and Omicron Waves: a prospective, observational, cohort study

Costanza Di Chiara*, Riccardo Boracchini, Giulia Sturniolo, Alessia Barbieri, Paola Costenaro, Sandra Cozzani[1], Marica De Pieri, Cecilia Liberati, Annachiara Zin, Andrea Padoan, Francesco Bonfante, Fatima Kakkar, Anna Cantarutti, Daniele Donà†, Carlo Giaquinto†

†These authors contributed equally to this work and share last authorship

*** Correspondence:** Costanza Di Chiara, costanza.dichiara@phd.unipd.it

1 Supplementary Figures

Supplementary Figure 1. Number of COVID-19 pediatric cases enrolled between April 2020 and December 2022 at the COVID-19 Family Cluster Follow-up Clinic (CovFC) of our institution. In gray boxes are presented the criteria for the definition of the baseline of infection for COVID-19 cases.

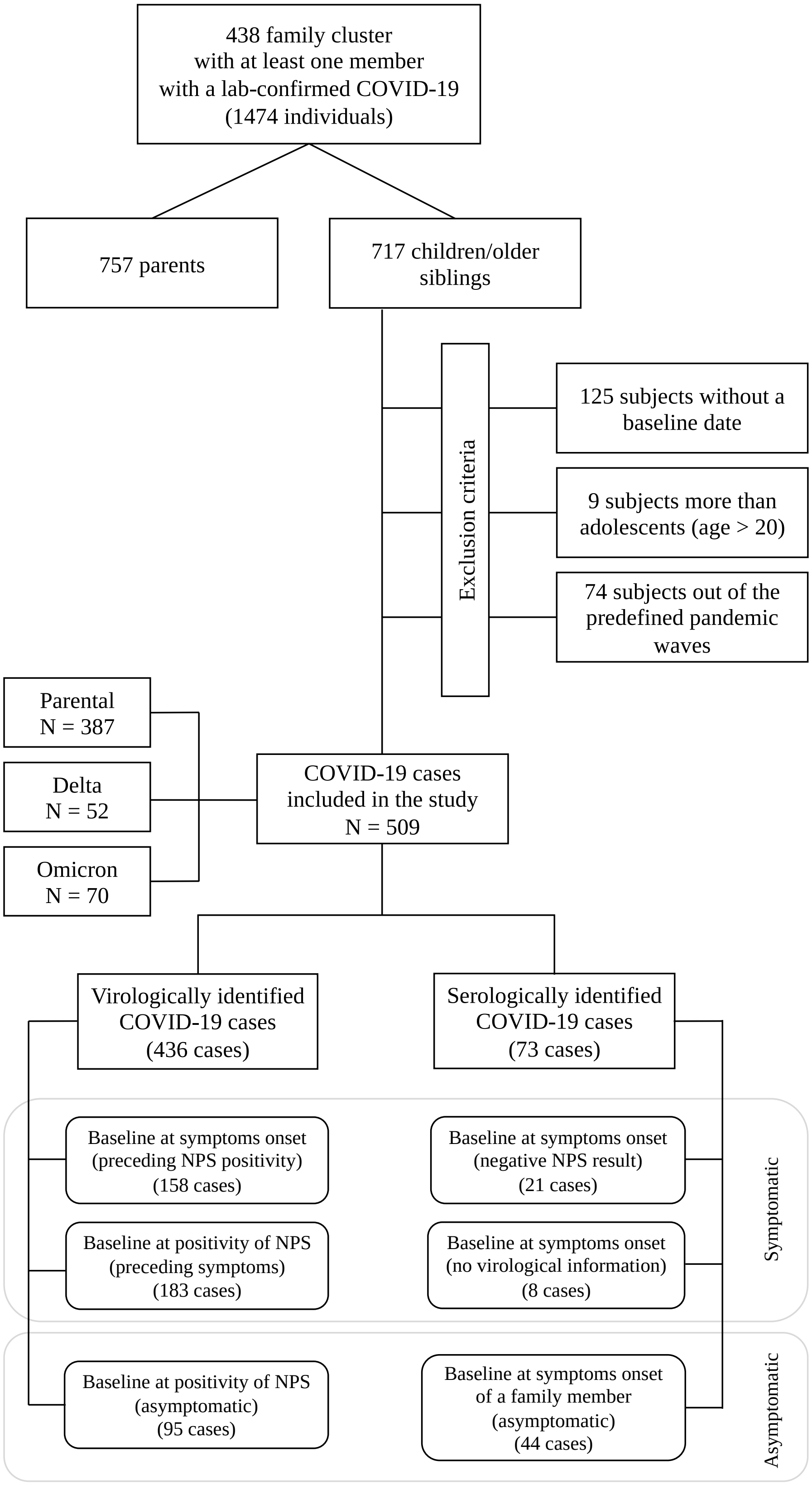
438 family cluster with at least one member with a lab-confirmed COVID-19 (1474 individuals)
757 parents
717 children/older siblings
Exclusion criteria
125 subjects without a baseline date
9 subjects more than adolescents (age > 20)
74 subjects out of the predefined pandemic waves
Parental N = 387
Delta N = 52
Omicron N = 70
COVID-19 cases included in the study N = 509
Virologically identified COVID-19 cases (436 cases)
Serologically identified COVID-19 cases (73 cases)
Baseline at symptoms onset (preceding NPS positivity) (158 cases)
Baseline at positivity of NPS (preceding symptoms) (183 cases)
Baseline at positivity of NPS (asymptomatic) (95 cases)
Baseline at symptoms onset (negative NPS result) (21 cases)
Baseline at symptoms onset (no virological information) (8 cases)
Baseline at symptoms onset of a family member (asymptomatic) (44 cases)
Symptomatic
Asymptomatic

2 Supplementary Tables

Supplementary Table 1. Differences in clinical manifestations of SARS-CoV-2 infection between children who had previously received at least one dose of COVID-19 vaccine and those children who did not.

	Unvaccinated (N = 47)	Vaccinated (N = 23)	P - Value
Symptoms, N (%)			
Rhinitis	17 (36.17)	9 (39.13)	0.8097
Cough	17 (36.17)	7 (30.43)	0.6349
Dyspnea	1 (2.13)	1 (4.35)	0.4476
Fever	39 (82.98)	14 (60.87)	0.0427
Otalgia	0 (0)	0 (0)	-
Myalgia	3 (6.38)	2 (8.7)	0.339
Arthralgia	0 (0)	2 (8.7)	0.1048
Sore throat	6 (12.77)	4 (17.39)	0.2397
Hyposmia and/or ageusia	2 (4.26)	1 (4.35)	0.4542
Conjunctivitis	1 (2.13)	0 (0)	0.6714
Fatigue	10 (21.28)	5 (21.74)	0.2415
Headache	10 (21.28)	6 (26.09)	0.6526
Alteration of consciousness	0 (0)	0 (0)	-
Abdominal pain	1 (2.13)	0 (0)	0.6714
Nausea/vomiting	4 (8.51)	1 (4.35)	0.339
Diarrhea	4 (8.51)	0 (0)	0.1945
Poor feeding	3 (6.38)	1 (4.35)	0.4067
Lymphadenopathy	0 (0)	0 (0)	-
Skin rash	0 (0)	0 (0)	-
Pneumonia	1 (2.13)	0 (0)	0.6714
Other symptoms	5 (10.64)	0 (0)	0.1267
Number of symptoms, N (%)			
0	1 (2.13)	1 (4.35)	0.0175
1	9 (19.15)	5 (21.74)	
2	13 (27.66)	8 (34.78)	
≥ 3	24 (51.06)	9 (39.13)	
Duration of symptoms			
days	2 (1 - 4)	3 (1 - 4)	0.7403

Supplementary Table 2. Clinical features of SARS-CoV-2 infection among different age groups (0-2 months, 3-11 months, 1-4 years, 5-11 years, and 12-20 years).

	0 - 2 months (N=13)	3 - 11 months (N=36)	1 - 4 years (N=83)	5 - 11 years (N=248)	12 - 20 years (N=129)
Rhinitis	5 (38.46)	7 (19.44)	24 (28.92)	40 (16.13)	29 (22.48)
Fever	10 (76.92)	22 (61.11)	45 (54.22)	99 (39.92)	62 (48.06)
Myalgia	2 (15.38)	5 (13.89)	20 (24.1)	28 (11.29)	20 (15.5)
Arthralgia	0 (0)	0 (0)	2 (2.41)	6 (2.42)	7 (5.43)
Sore throat	N/A	N/A	2 (2.41)	13 (5.24)	13 (10.08)
Hyposmia and/or ageusia	N/A	N/A	0 (0)	20 (8.06)	26 (20.16)
Fatigue	1 (7.69)	1 (2.78)	8 (9.64)	43 (17.34)	26 (20.16)
Headache	N/A	N/A	2 (2.41)	49 (19.76)	29 (22.48)
Abdominal pain	0 (0)	0 (0)	3 (3.61)	10 (4.03)	2 (1.55)
Nausea/vomiting	0 (0)	1 (2.78)	2 (2.41)	12 (4.84)	8 (6.2)
Diarrhea	0 (0)	7 (19.44)	8 (9.64)	15 (6.05)	9 (6.98)
Poor feeding	2 (15.38)	4 (11.11)	5 (6.02)	7 (2.82)	2 (1.55)
Number of symptoms					
0	1 (7.69)	8 (22.22)	20 (24.1)	82 (33.06)	28 (21.71)
1	6 (46.15)	11 (30.56)	24 (28.92)	60 (24.19)	33 (25.58)
2	3 (23.08)	10 (27.78)	16 (19.28)	46 (18.55)	29 (22.48)
≥ 3	3 (23.08)	7 (19.44)	23 (27.71)	60 (24.19)	39 (30.23)
Duration of symptoms, days	2 (1 - 5)	3 (2 - 7)	3 (1 - 6)	3 (1 - 5)	3 (1 - 7)

N/A = not assessed.

Supplementary Table 3. Clinical features of COVID-19 among different age groups (0-2, 3-4, 5-11, and 12-20 years), overall (N=509) and stratified according to the variant of concern (VOC) (Parental=387, Delta=52, Omicron=70).

		0 - 2 years	3 - 4 years	5 - 11 years	12 - 20 years	P-value
Rhinitis – Overall, N (%)		25 (29.41)	15 (23.44)	36 (15.58)	29 (22.48)	0.0426
	Parental	15 (22.39)	9 (19.15)	16 (9.7)	22 (20.37)	0.0323
	Delta	3 (37.5)	4 (57.14)	8 (27.59)	2 (25)	0.0107
	Omicron	7 (70)	2 (20)	12 (32.43)	5 (38.46)	0.0012
Cough - Overall, N (%)		17 (20)	12 (18.75)	26 (11.26)	20 (15.5)	0.173
	Parental	9 (13.43)	7 (14.89)	9 (5.45)	12 (11.11)	0.1011
	Delta	5 (62.5)	1 (14.29)	5 (17.24)	3 (37.5)	0.0015
	Omicron	3 (30)	4 (40)	12 (32.43)	5 (38.46)	0.0171
Dyspnea - Overall, N (%)		2 (2.35)	0 (0)	2 (0.87)	3 (2.33)	0.0197
	Parental	2 (2.99)	0 (0)	2 (1.21)	1 (0.93)	0.0458
	Delta	0 (0)	0 (0)	0 (0)	0 (0)	-
	Omicron	0 (0)	0 (0)	0 (0)	2 (15.38)	0.0323
Fever - Overall, N (%)		54 (63.53)	31 (48.44)	91 (39.39)	62 (48.06)	0.002
	Parental	41 (61.19)	18 (38.3)	51 (30.91)	48 (44.44)	0.0003
	Delta	4 (50)	5 (71.43)	13 (44.83)	5 (62.5)	0.0117
	Omicron	9 (90)	8 (80)	27 (72.97)	9 (69.23)	0.0142
Otalgia - Overall, N (%)		1 (1.18)	1 (1.56)	0 (0)	0 (0)	0.0421
	Parental	1 (1.49)	0 (0)	0 (0)	0 (0)	0.1731
	Delta	0 (0)	1 (14.29)	0 (0)	0 (0)	0.1346
	Omicron	0 (0)	0 (0)	0 (0)	0 (0)	-
Myalgia - Overall, N (%)		0 (0)	1 (1.56)	10 (4.33)	10 (7.75)	0.0002
	Parental	0 (0)	1 (2.13)	6 (3.64)	7 (6.48)	0.0022
	Delta	0 (0)	0 (0)	1 (3.45)	1 (12.5)	0.175
	Omicron	0 (0)	0 (0)	3 (8.11)	2 (15.38)	0.0501
Arthralgia - Overall, N (%)		0 (0)	3 (4.69)	5 (2.16)	7 (5.43)	0.0009
	Parental	0 (0)	3 (6.38)	5 (3.03)	5 (4.63)	0.0030
	Delta	0 (0)	0 (0)	0 (0)	0 (0)	-
	Omicron	0 (0)	0 (0)	0 (0)	2 (15.38)	0.0323
Sore throat - Overall, N (%)		1 (1.18)	1 (1.56)	13 (5.63)	13 (10.08)	< 0.0001
	Parental	1 (1.49)	0 (0)	6 (3.64)	10 (9.26)	0.0003
	Delta	0 (0)	0 (0)	0 (0)	1 (12.5)	0.1538
	Omicron	0 (0)	1 (10)	7 (18.92)	2 (15.38)	0.0202
Hyposmia and/or ageusia - Overall, N (%)		0 (0)	1 (1.56)	19 (8.23)	26 (20.16)	< 0.0001
	Parental	0 (0)	1 (2.13)	16 (9.7)	21 (19.44)	< 0.0001
	Delta	0 (0)	0 (0)	2 (6.9)	3 (37.5)	0.0087
	Omicron	0 (0)	0 (0)	1 (2.7)	2 (15.38)	0.0527
Conjunctivitis - Overall, N (%)		2 (2.35)	2 (3.13)	5 (2.16)	3 (2.33)	0.0238
	Parental	2 (2.99)	1 (2.13)	4 (2.42)	3 (2.78)	0.0342
	Delta	0 (0)	0 (0)	1 (3.45)	0 (0)	0.5577
	Omicron	0 (0)	1 (10)	0 (0)	0 (0)	0.1429
Fatigue - Overall, N (%)		5 (5.88)	8 (12.5)	40 (17.32)	26 (20.16)	0.0274
	Parental	4 (5.97)	3 (6.38)	27 (16.36)	21 (19.44)	0.0274

Delta	0 (0)	2 (28.57)	5 (17.24)	2 (25)	0.019
Omicron	1 (10)	3 (30)	8 (21.62)	3 (23.08)	0.0184
Headache - Overall, N (%)	0 (0)	3 (4.69)	48 (20.78)	29 (22.48)	< 0.0001
Parental	0 (0)	3 (6.38)	29 (17.58)	24 (22.22)	0.0001
Delta	0 (0)	0 (0)	6 (20.69)	2 (25)	0.0177
Omicron	0 (0)	0 (0)	13 (35.14)	3 (23.08)	0.0004
Alteration of consciousness - Overall, N (%)	0 (0)	0 (0)	0 (0)	0 (0)	-
Parental	0 (0)	0 (0)	0 (0)	0 (0)	-
Delta	0 (0)	0 (0)	0 (0)	0 (0)	-
Omicron	0 (0)	0 (0)	0 (0)	0 (0)	-
Abdominal pain - Overall, N (%)	0 (0)	4 (6.25)	9 (3.9)	2 (1.55)	0.0009
Parental	0 (0)	3 (6.38)	8 (4.85)	2 (1.85)	0.0019
Delta	0 (0)	0 (0)	1 (3.45)	0 (0)	0.5577
Omicron	0 (0)	1 (10)	0 (0)	0 (0)	0.1429
Nausea/vomiting - Overall, N (%)	3 (3.53)	1 (1.56)	11 (4.76)	8 (6.2)	0.0045
Parental	3 (4.48)	0 (0)	6 (3.64)	7 (6.48)	0.0039
Delta	0 (0)	0 (0)	2 (6.9)	0 (0)	0.3062
Omicron	0 (0)	1 (10)	3 (8.11)	1 (7.69)	0.0835
Diarrhea - Overall, N (%)	12 (14.12)	5 (7.81)	13 (5.63)	9 (6.98)	0.0918
Parental	9 (13.43)	4 (8.51)	11 (6.67)	7 (6.48)	0.3291
Delta	0 (0)	1 (14.29)	2 (6.9)	1 (12.5)	0.084
Omicron	3 (30)	0 (0)	0 (0)	1 (7.69)	0.0017
Poor feeding - Overall, N (%)	7 (8.24)	5 (7.81)	6 (2.6)	2 (1.55)	0.0002
Parental	6 (8.96)	4 (8.51)	3 (1.82)	2 (1.85)	0.0002
Delta	0 (0)	0 (0)	1 (3.45)	0 (0)	0.5577
Omicron	1 (10)	1 (10)	2 (5.41)	0 (0)	0.0726
Lymphadenopathy - Overall, N (%)	0 (0)	0 (0)	1 (0.43)	0 (0)	0.4538
Parental	0 (0)	0 (0)	1 (0.61)	0 (0)	0.4264
Delta	0 (0)	0 (0)	0 (0)	0 (0)	-
Omicron	0 (0)	0 (0)	0 (0)	0 (0)	-
Skin rash - Overall, N (%)	1 (1.18)	3 (4.69)	4 (1.73)	4 (3.1)	0.0081
Parental	1 (1.49)	2 (4.26)	4 (2.42)	4 (3.7)	0.0183
Delta	0 (0)	1 (14.29)	0 (0)	0 (0)	0.1346
Omicron	0 (0)	0 (0)	0 (0)	0 (0)	-
Pneumonia - Overall, N (%)	0 (0)	0 (0)	2 (0.87)	1 (0.78)	0.1568
Parental	0 (0)	0 (0)	2 (1.21)	0 (0)	0.1811
Delta	0 (0)	0 (0)	0 (0)	0 (0)	-
Omicron	0 (0)	0 (0)	0 (0)	1 (7.69)	0.1857
Other symptoms - Overall, N (%)	7 (8.24)	5 (7.81)	10 (4.33)	13 (10.08)	0.192
Parental	4 (5.97)	4 (8.51)	9 (5.45)	11 (10.19)	0.0027
Delta	1 (12.5)	0 (0)	0 (0)	1 (12.5)	0.0483
Omicron	2 (20)	1 (10)	1 (2.7)	1 (7.69)	0.0179
Number of symptoms - Overall, N (%)					
0	15 (17.65)	20 (31.25)	76 (32.9)	28 (21.71)	0.1426
1	30 (35.29)	13 (20.31)	58 (25.11)	33 (25.58)	
2	19 (22.35)	14 (21.88)	42 (18.18)	29 (22.48)	
≥ 3	21 (24.71)	17 (26.56)	55 (23.81)	39 (30.23)	

Parental					
0	12 (17.91)	19 (40.43)	67 (40.61)	26 (24.07)	0.008
1	27 (40.3)	8 (17.02)	43 (26.06)	32 (29.63)	
2	16 (23.88)	9 (19.15)	22 (13.33)	22 (20.37)	
≥ 3	12 (17.91)	11 (23.4)	33 (20)	28 (25.93)	
Delta					
0	3 (37.5)	0 (0)	8 (27.59)	2 (25)	< 0.0001
1	1 (12.5)	3 (42.86)	6 (20.69)	0 (0)	
2	1 (12.5)	2 (28.57)	9 (31.03)	2 (25)	
≥ 3	3 (37.5)	2 (28.57)	6 (20.69)	4 (50)	
Omicron					
0	0 (0)	1 (10)	1 (2.7)	0 (0)	< 0.0001
1	2 (20)	2 (20)	9 (24.32)	1 (7.69)	
2	2 (20)	3 (30)	11 (29.73)	5 (38.46)	
≥ 3	6 (60)	4 (40)	16 (43.24)	7 (53.85)	
Duration of symptoms, days					
Overall	3 (1 - 6)	3 (1 - 7)	2.5 (1 - 5)	3 (1 - 7)	0.4714
parental	3 (1 - 7)	3 (1 - 5)	3 (1 - 5)	3 (1 - 7)	0.9248
Delta	5 (3 - 7)	7 (7 - 19)	2 (1 - 2)	7 (2 - 13)	0.1442
Omicron	2 (0 - 3.5)	2 (1 - 3)	2 (1 - 4)	6 (1 - 9)	0.3136

Supplementary Table 4. Clinical features of SARS-CoV-2 infection in children with at least one underlying disease compare with healthy subjects, overall, and stratified according to the variant of concern (VOC).

	Overall (N=509)		P-value	Parental (N = 387)		Delta (N = 52)		Omicron (N = 70)	
Comorbidities	0	At least 1		0	At least 1	0	At least 1	0	At least 1
***Symptoms*, N (%)**									
Rhinitis	73 (18.72)	32 (26.89)	0.0538	47 (15.51)	15 (17.86)	10 (24.39)	7 (63.64)	16 (34.78)	10 (41.67)
Cough	48 (12.31)	27 (22.69)	0.0052	24 (7.92)	13 (15.48)	11 (26.83)	3 (27.27)	13 (28.26)	11 (45.83)
Dyspnea	3 (0.77)	4 (3.36)	0.0462	3 (0.99)	2 (2.38)	0 (0)	0 (0)	0 (0)	2 (8.33)
Fever	174 (44.62)	64 (53.78)	0.0794	121 (39.93)	37 (44.05)	20 (48.78)	7 (63.64)	33 (71.74)	20 (83.33)
Otalgia	2 (0.51)	0 (0)	0.5867	1 (0.33)	0 (0)	1 (2.44)	0 (0)	0 (0)	0 (0)
Myalgia	14 (3.59)	7 (5.88)	0.107	11 (3.63)	3 (3.57)	2 (4.88)	0 (0)	1 (2.17)	4 (16.67)
Arthralgia	11 (2.82)	4 (3.36)	0.2212	10 (3.3)	3 (3.57)	0 (0)	0 (0)	1 (2.17)	1 (4.17)
Sore throat	22 (5.64)	6 (5.04)	0.8019	14 (4.62)	3 (3.57)	1 (2.44)	0 (0)	7 (15.22)	3 (12.5)
Hyposmia and/or ageusia	33 (8.46)	13 (10.92)	0.4121	29 (9.57)	9 (10.71)	4 (9.76)	1 (9.09)	0 (0)	3 (12.5)
Conjunctivitis	9 (2.31)	3 (2.52)	0.259	8 (2.64)	2 (2.38)	0 (0)	1 (9.09)	1 (2.17)	0 (0)
Fatigue	65 (16.67)	14 (11.76)	0.1961	46 (15.18)	9 (10.71)	7 (17.07)	2 (18.18)	12 (26.09)	3 (12.5)
Headache	62 (15.9)	18 (15.13)	0.8396	44 (14.52)	12 (14.29)	5 (12.2)	3 (27.27)	13 (28.26)	3 (12.5)
Alteration of consciousness	0 (0)	0 (0)	-	0 (0)	0 (0)	0 (0)	0 (0)	0 (0)	0 (0)
Abdominal pain	11 (2.82)	4 (3.36)	0.2212	9 (2.97)	4 (4.76)	1 (2.44)	0 (0)	1 (2.17)	0 (0)
Nausea/vomiting	15 (3.85)	8 (6.72)	0.186	11 (3.63)	5 (5.95)	2 (4.88)	0 (0)	2 (4.35)	3 (12.5)
Diarrhea	28 (7.18)	11 (9.24)	0.4587	22 (7.26)	9 (10.71)	3 (7.32)	1 (9.09)	3 (6.52)	1 (4.17)
Poor feeding	16 (4.1)	4 (3.36)	0.2074	12 (3.96)	3 (3.57)	1 (2.44)	0 (0)	3 (6.52)	1 (4.17)
Lymphadenopathy	1 (0.26)	0 (0)	0.7662	1 (0.33)	0 (0)	0 (0)	0 (0)	0 (0)	0 (0)
Skin rash	9 (2.31)	3 (2.52)	0.2590	8 (2.64)	3 (3.57)	1 (2.44)	0 (0)	0 (0)	0 (0)
Pneumonia	0 (0)	3 (2.52)	0.0125	0 (0)	2 (2.38)	0 (0)	0 (0)	0 (0)	1 (4.17)
Other symptoms	23 (5.9)	12 (10.08)	0.1142	20 (6.6)	8 (9.52)	1 (2.44)	1 (9.09)	2 (4.35)	3 (12.5)
Number of symptoms, N (%)									
0	117 (30)	22 (18.49)	0.0906	102 (33.66)	22 (26.19)	13 (31.71)	0 (0)	2 (4.35)	0 (0)
1	98 (25.13)	36 (30.25)		83 (27.39)	27 (32.14)	7 (17.07)	3 (27.27)	8 (17.39)	6 (25)
2	79 (20.26)	25 (21.01)		53 (17.49)	16 (19.05)	10 (24.39)	4 (36.36)	16 (34.78)	5 (20.83)
≥ 3	96 (24.62)	36 (30.25)		65 (21.45)	19 (22.62)	11 (26.83)	4 (36.36)	20 (43.48)	13 (54.17)
Duration of symptoms									
days	3 (1 - 5)	3 (1 - 7)	0.3302	3 (1 - 5)	2 (1 - 6.5)	2 (1.5 - 7)	7 (2 - 12)	1.5 (0.5 - 3)	4 (2 - 7)

Supplementary Table 5. Impact on COVID-19 clinical manifestations of influenza and rotavirus immunization status.

	Flu Vaccine*		P-value	Rotavirus**		P-value
	No (N = 327)	Yes (N = 59)		No (N = 148)	Yes (N = 353)	
Symptoms, N (%)						
Rhinitis	58 (17.74)	10 (16.95)	0.8838	22 (14.86)	80 (22.66)	0.048
Cough	35 (10.7)	10 (16.95)	0.1688	23 (15.54)	51 (14.45)	0.7531
Dyspnea	5 (1.53)	0 (0)	0.4343	2 (1.35)	4 (1.13)	0.3246
Fever	144 (44.04)	24 (40.68)	0.632	69 (46.62)	164 (46.46)	0.9734
Otalgia	2 (0.61)	0 (0)	0.7173	1 (0.68)	1 (0.28)	0.4171
Myalgia	14 (4.28)	3 (5.08)	0.2438	3 (2.03)	18 (5.1)	0.1175
Arthralgia	11 (3.36)	0 (0)	0.1571	2 (1.35)	13 (3.68)	0.0949
Sore throat	15 (4.59)	4 (6.78)	0.1794	7 (4.73)	21 (5.95)	0.5878
Hyposmia and/or ageusia	33 (10.09)	6 (10.17)	0.9854	11 (7.43)	35 (9.92)	0.38
Conjunctivitis	9 (2.75)	0 (0)	0.2209	3 (2.03)	8 (2.27)	0.261
Fatigue	48 (14.68)	9 (15.25)	0.9087	18 (12.16)	61 (17.28)	0.1515
Headache	45 (13.76)	8 (13.56)	0.9669	20 (13.51)	60 (17)	0.3315
Alteration of consciousness	0 (0)	0 (0)	-	0 (0)	0 (0)	-
Abdominal pain	11 (3.36)	1 (1.69)	0.2966	5 (3.38)	10 (2.83)	0.2067
Nausea/vomiting	13 (3.98)	2 (3.39)	0.2887	8 (5.41)	15 (4.25)	0.5727
Diarrhea	25 (7.65)	2 (3.39)	0.1262	15 (10.14)	24 (6.8)	0.2035
Poor feeding	10 (3.06)	3 (5.08)	0.1971	4 (2.7)	15 (4.25)	0.4083
Lymphadenopathy	1 (0.31)	0 (0)	0.8472	0 (0)	1 (0.28)	0.7046
Skin rash	9 (2.75)	3 (5.08)	0.1783	7 (4.73)	5 (1.42)	0.0259
Pneumonia	1 (0.31)	0 (0)	0.8472	2 (1.35)	1 (0.28)	0.1843
Other symptoms	17 (5.2)	5 (8.47)	0.1327	12 (8.11)	22 (6.23)	0.4463
Number of symptoms, N (%)						
0	104 (31.80)	18 (30.51)	0.2463	45 (30.41)	93 (26.35)	0.7144
1	86 (26.30)	14 (23.73)		39 (26.35)	91 (25.78)	
2	59 (18.04)	17 (28.81)		30 (20.27)	73 (20.68)	
≥ 3	78 (23.85)	10 (16.95)		34 (22.97)	96 (27.20)	
Duration of symptoms						
days	3 (1 - 7)	2 (0 - 4)	0.0955	3 (1 - 7)	3 (1 - 6)	0.9879

* Patients aged at least 6 months, 96 missing values are handled in the analysis; **Patients aged more than 61 days
